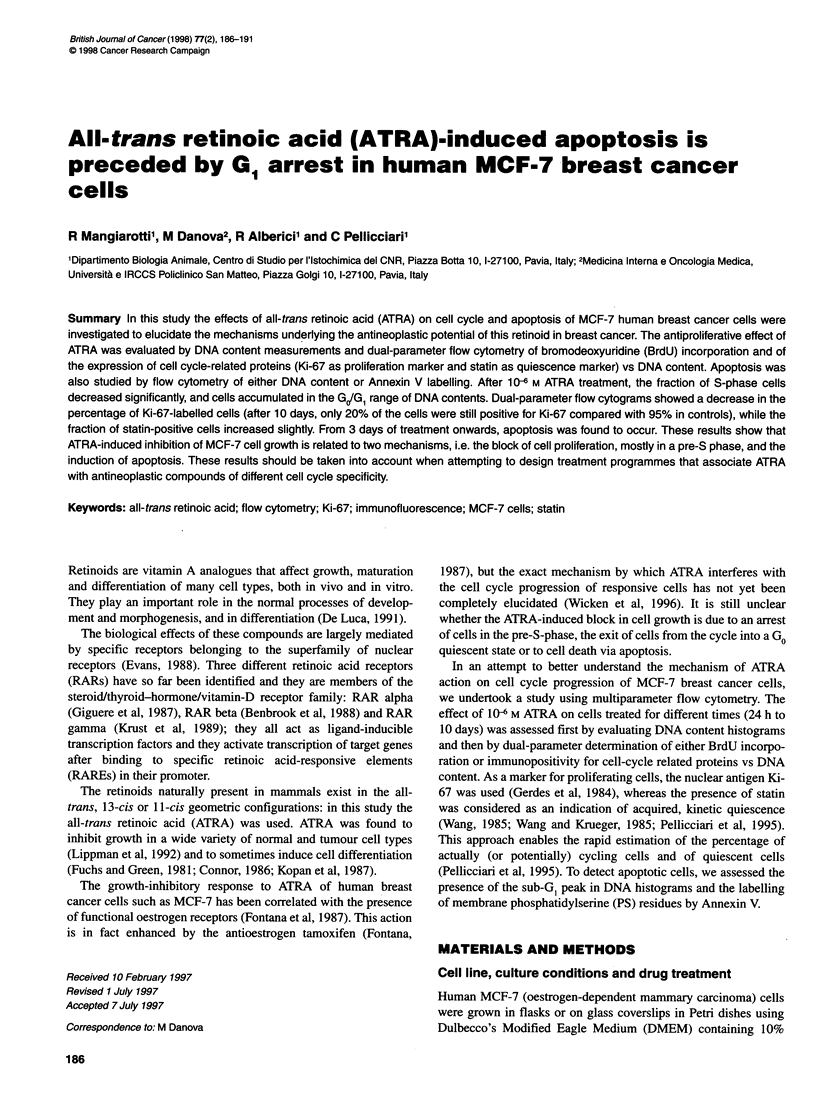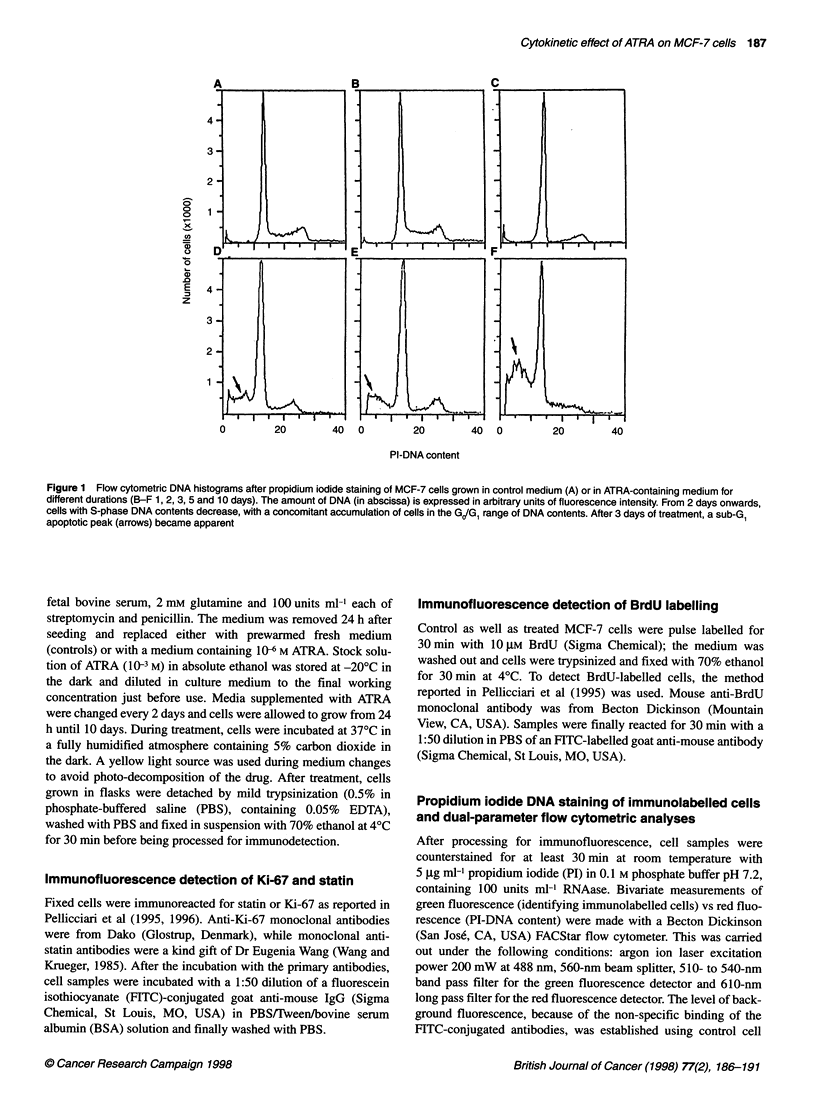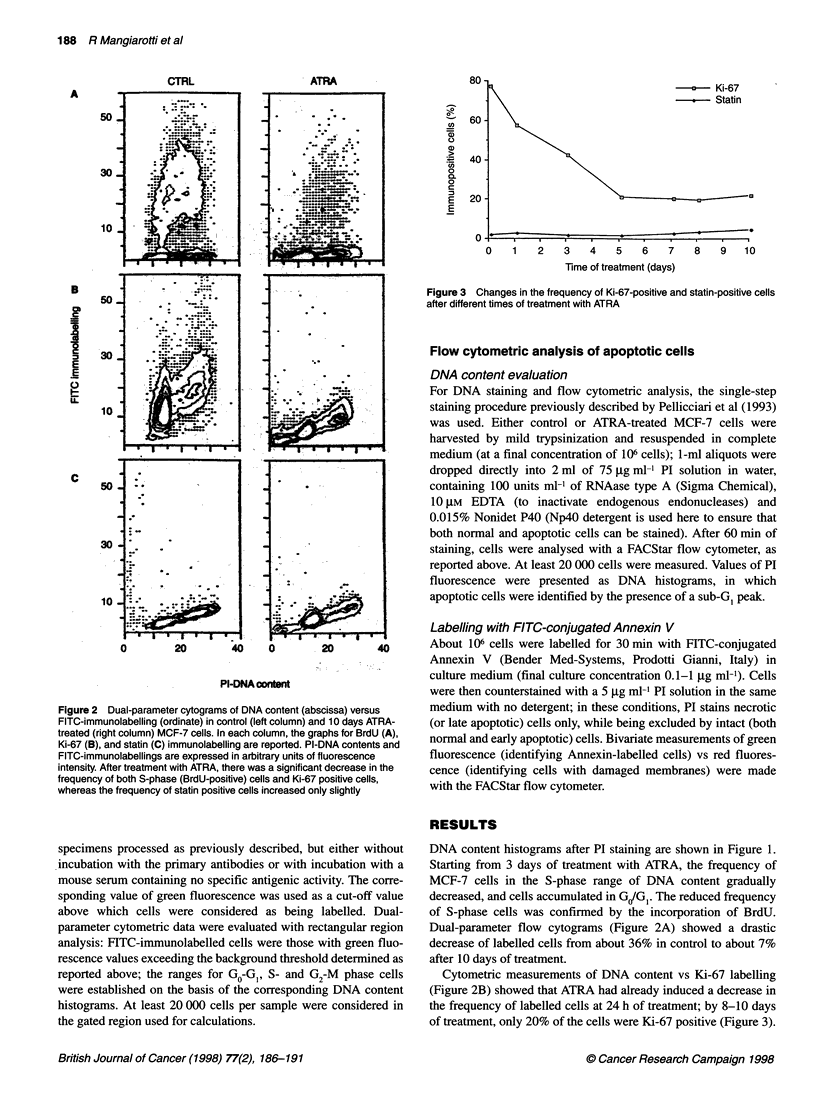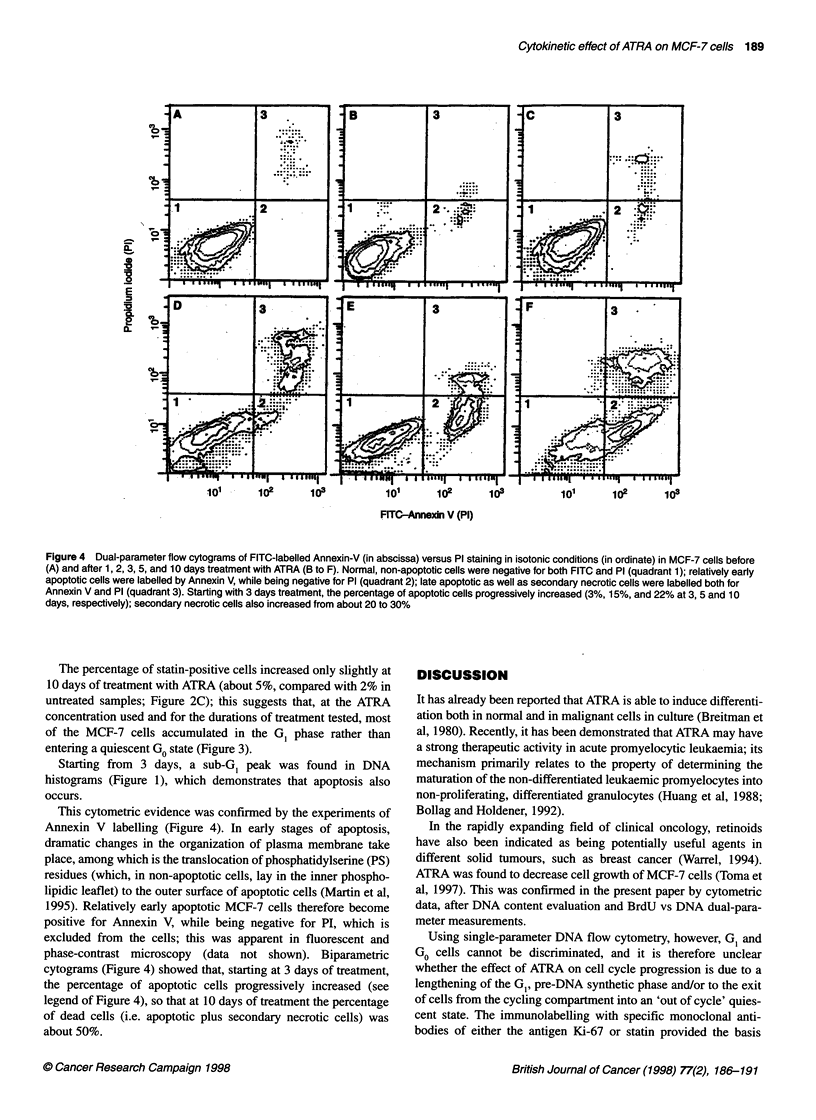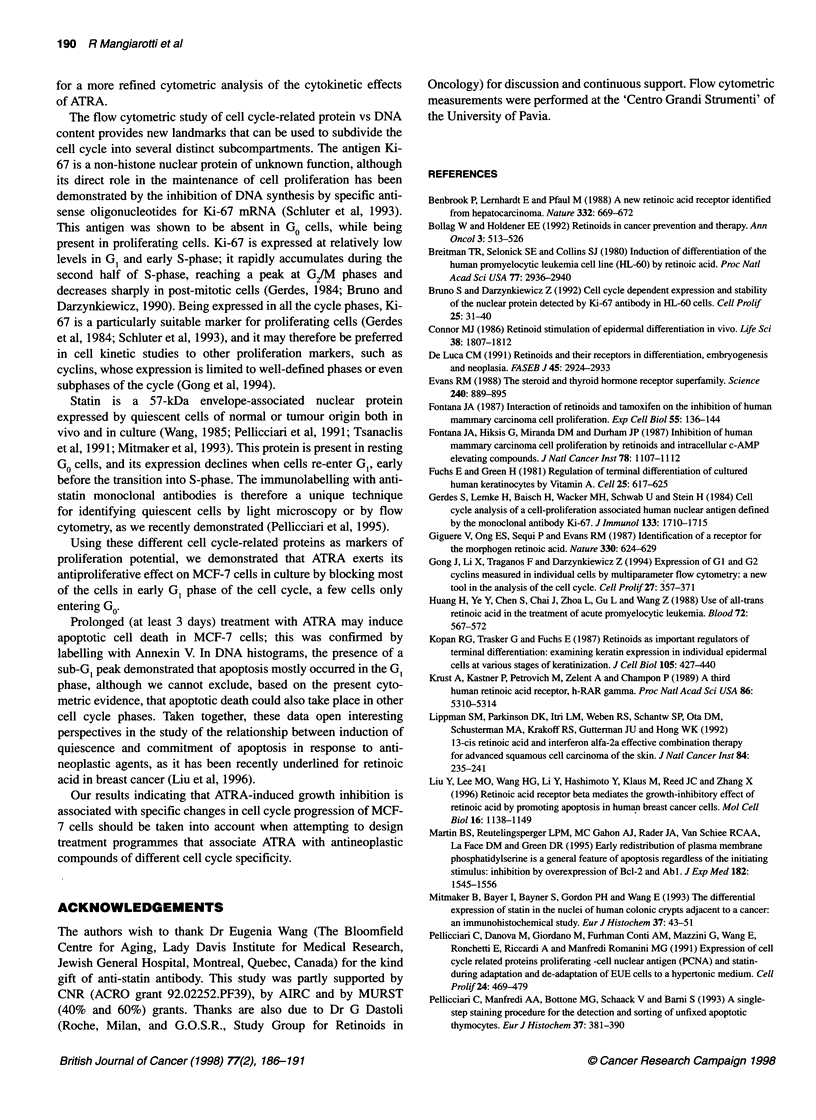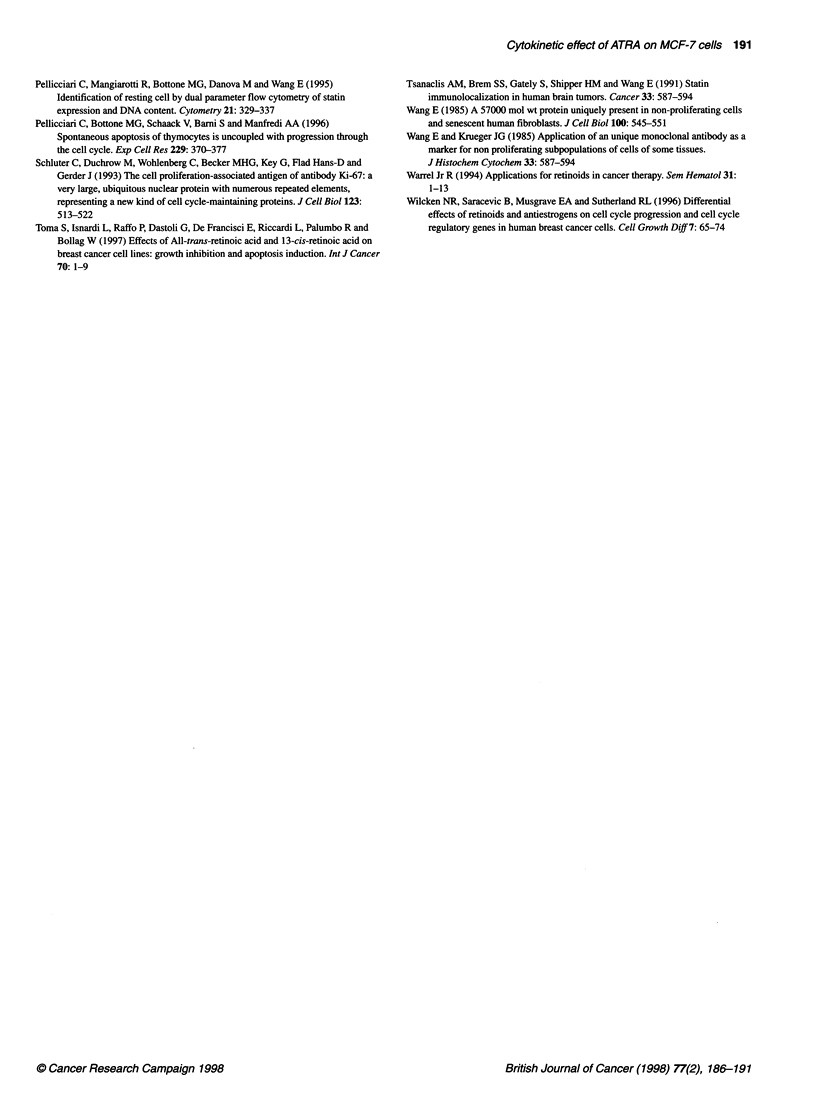# All-trans retinoic acid (ATRA)-induced apoptosis is preceded by G1 arrest in human MCF-7 breast cancer cells.

**DOI:** 10.1038/bjc.1998.32

**Published:** 1998

**Authors:** R. Mangiarotti, M. Danova, R. Alberici, C. Pellicciari

**Affiliations:** Dipartimento Biologia Animale, Centro di Studio per l'Istochimica del CNR, Pavia, Italy.

## Abstract

In this study the effects of all-trans retinoic acid (ATRA) on cell cycle and apoptosis of MCF-7 human breast cancer cells were investigated to elucidate the mechanisms underlying the antineoplastic potential of this retinoid in breast cancer. The antiproliferative effect of ATRA was evaluated by DNA content measurements and dual-parameter flow cytometry of bromodeoxyuridine (BrdU) incorporation and of the expression of cell cycle-related proteins (Ki-67 as proliferation marker and statin as quiescence marker) vs DNA content. Apoptosis was also studied by flow cytometry of either DNA content or Annexin V labelling. After 10(-6) M ATRA treatment, the fraction of S-phase cells decreased significantly, and cells accumulated in the G0/G1 range of DNA contents. Dual-parameter flow cytograms showed a decrease in the percentage of Ki-67-labelled cells (after 10 days, only 20% of the cells were still positive for Ki-67 compared with 95% in controls), while the fraction of statin-positive cells increased slightly. From 3 days of treatment onwards, apoptosis was found to occur. These results show that ATRA-induced inhibition of MCF-7 cell growth is related to two mechanisms, i.e. the block of cell proliferation, mostly in a pre-S phase, and the induction of apoptosis. These results should be taken into account when attempting to design treatment programmes that associate ATRA with antineoplastic compounds of different cell cycle specificity.


British Journal of Cancer (1998) 77(2), 186–191
© 1998 Cancer Research Campaign

### All-*trans* retinoic acid (ATRA)-induced apoptosis is preceded by $G_1$ arrest in human MCF-7 breast cancer cells

**R Mangiarotti[1], M Danova[2], R Alberici[1] and C Pellicciari[1]**

[1]Dipartimento Biologia Animale, Centro di Studio per l'Istochimica del CNR, Piazza Botta 10, I-27100, Pavia, Italy; [2]Medicina Interna e Oncologia Medica, Università e IRCCS Policlinico San Matteo, Piazza Golgi 10, I-27100, Pavia, Italy

**Summary** In this study the effects of all-*trans* retinoic acid (ATRA) on cell cycle and apoptosis of MCF-7 human breast cancer cells were investigated to elucidate the mechanisms underlying the antineoplastic potential of this retinoid in breast cancer. The antiproliferative effect of ATRA was evaluated by DNA content measurements and dual-parameter flow cytometry of bromodeoxyuridine (BrdU) incorporation and of the expression of cell cycle-related proteins (Ki-67 as proliferation marker and statin as quiescence marker) vs DNA content. Apoptosis was also studied by flow cytometry of either DNA content or Annexin V labelling. After $10^{-6}$ M ATRA treatment, the fraction of S-phase cells decreased significantly, and cells accumulated in the $G_0/G_1$ range of DNA contents. Dual-parameter flow cytograms showed a decrease in the percentage of Ki-67-labelled cells (after 10 days, only 20% of the cells were still positive for Ki-67 compared with 95% in controls), while the fraction of statin-positive cells increased slightly. From 3 days of treatment onwards, apoptosis was found to occur. These results show that ATRA-induced inhibition of MCF-7 cell growth is related to two mechanisms, i.e. the block of cell proliferation, mostly in a pre-S phase, and the induction of apoptosis. These results should be taken into account when attempting to design treatment programmes that associate ATRA with antineoplastic compounds of different cell cycle specificity.

**Keywords:** all-*trans* retinoic acid; flow cytometry; Ki-67; immunofluorescence; MCF-7 cells; statin

Retinoids are vitamin A analogues that affect growth, maturation and differentiation of many cell types, both in vivo and in vitro. They play an important role in the normal processes of development and morphogenesis, and in differentiation (De Luca, 1991).

The biological effects of these compounds are largely mediated by specific receptors belonging to the superfamily of nuclear receptors (Evans, 1988). Three different retinoic acid receptors (RARs) have so far been identified and they are members of the steroid/thyroid–hormone/vitamin-D receptor family: RAR alpha (Giguere et al, 1987), RAR beta (Benbrook et al, 1988) and RAR gamma (Krust et al, 1989); they all act as ligand-inducible transcription factors and they activate transcription of target genes after binding to specific retinoic acid-responsive elements (RAREs) in their promoter.

The retinoids naturally present in mammals exist in the all-*trans*, 13-*cis* or 11-*cis* geometric configurations: in this study the all-*trans* retinoic acid (ATRA) was used. ATRA was found to inhibit growth in a wide variety of normal and tumour cell types (Lippman et al, 1992) and to sometimes induce cell differentiation (Fuchs and Green, 1981; Connor, 1986; Kopan et al, 1987).

The growth-inhibitory response to ATRA of human breast cancer cells such as MCF-7 has been correlated with the presence of functional oestrogen receptors (Fontana et al, 1987). This action is in fact enhanced by the antioestrogen tamoxifen (Fontana, 1987), but the exact mechanism by which ATRA interferes with the cell cycle progression of responsive cells has not yet been completely elucidated (Wicken et al, 1996). It is still unclear whether the ATRA-induced block in cell growth is due to an arrest of cells in the pre-S-phase, the exit of cells from the cycle into a $G_0$ quiescent state or to cell death via apoptosis.

In an attempt to better understand the mechanism of ATRA action on cell cycle progression of MCF-7 breast cancer cells, we undertook a study using multiparameter flow cytometry. The effect of $10^{-6}$ M ATRA on cells treated for different times (24 h to 10 days) was assessed first by evaluating DNA content histograms and then by dual-parameter determination of either BrdU incorporation or immunopositivity for cell-cycle related proteins vs DNA content. As a marker for proliferating cells, the nuclear antigen Ki-67 was used (Gerdes et al, 1984), whereas the presence of statin was considered as an indication of acquired, kinetic quiescence (Wang, 1985; Wang and Krueger, 1985; Pellicciari et al, 1995). This approach enables the rapid estimation of the percentage of actually (or potentially) cycling cells and of quiescent cells (Pellicciari et al, 1995). To detect apoptotic cells, we assessed the presence of the sub-$G_1$ peak in DNA histograms and the labelling of membrane phosphatidylserine (PS) residues by Annexin V.

*Received 10 February 1997*
*Revised 1 July 1997*
*Accepted 7 July 1997*

*Correspondence to:* M Danova

### MATERIALS AND METHODS

### Cell line, culture conditions and drug treatment

Human MCF-7 (oestrogen-dependent mammary carcinoma) cells were grown in flasks or on glass coverslips in Petri dishes using Dulbecco's Modified Eagle Medium (DMEM) containing 10%

**Figure 1** Flow cytometric DNA histograms after propidium iodide staining of MCF-7 cells grown in control medium (A) or in ATRA-containing medium for different durations (B–F 1, 2, 3, 5 and 10 days). The amount of DNA (in abscissa) is expressed in arbitrary units of fluorescence intensity. From 2 days onwards, cells with S-phase DNA contents decrease, with a concomitant accumulation of cells in the $G_0/G_1$ range of DNA contents. After 3 days of treatment, a sub-$G_1$ apoptotic peak (arrows) became apparent fetal bovine serum, 2 mM glutamine and 100 units $ml^{-1}$ each of streptomycin and penicillin. The medium was removed 24 h after seeding and replaced either with prewarmed fresh medium (controls) or with a medium containing $10^{-6}$ M ATRA. Stock solution of ATRA ($10^{-3}$ M) in absolute ethanol was stored at −20°C in the dark and diluted in culture medium to the final working concentration just before use. Media supplemented with ATRA were changed every 2 days and cells were allowed to grow from 24 h until 10 days. During treatment, cells were incubated at 37°C in a fully humidified atmosphere containing 5% carbon dioxide in the dark. A yellow light source was used during medium changes to avoid photo-decomposition of the drug. After treatment, cells grown in flasks were detached by mild trypsinization (0.5% in phosphate-buffered saline (PBS), containing 0.05% EDTA), washed with PBS and fixed in suspension with 70% ethanol at 4°C for 30 min before being processed for immunodetection.

### Immunofluorescence detection of Ki-67 and statin

Fixed cells were immunoreacted for statin or Ki-67 as reported in Pellicciari et al (1995, 1996). Anti-Ki-67 monoclonal antibodies were from Dako (Glostrup, Denmark), while monoclonal anti-statin antibodies were a kind gift of Dr Eugenia Wang (Wang and Krueger, 1985). After the incubation with the primary antibodies, cell samples were incubated with a 1:50 dilution of a fluorescein isothiocyanate (FITC)-conjugated goat anti-mouse IgG (Sigma Chemical, St Louis, MO, USA) in PBS/Tween/bovine serum albumin (BSA) solution and finally washed with PBS.

### Immunofluorescence detection of BrdU labelling

Control as well as treated MCF-7 cells were pulse labelled for 30 min with 10 μM BrdU (Sigma Chemical); the medium was washed out and cells were trypsinized and fixed with 70% ethanol for 30 min at 4°C. To detect BrdU-labelled cells, the method reported in Pellicciari et al (1995) was used. Mouse anti-BrdU monoclonal antibody was from Becton Dickinson (Mountain View, CA, USA). Samples were finally reacted for 30 min with a 1:50 dilution in PBS of an FITC-labelled goat anti-mouse antibody (Sigma Chemical, St Louis, MO, USA).

### Propidium iodide DNA staining of immunolabelled cells and dual-parameter flow cytometric analyses

After processing for immunofluorescence, cell samples were counterstained for at least 30 min at room temperature with 5 μg $ml^{-1}$ propidium iodide (PI) in 0.1 M phosphate buffer pH 7.2, containing 100 units $ml^{-1}$ RNAase. Bivariate measurements of green fluorescence (identifying immunolabelled cells) vs red fluorescence (PI-DNA content) were made with a Becton Dickinson (San José, CA, USA) FACStar flow cytometer. This was carried out under the following conditions: argon ion laser excitation power 200 mW at 488 nm, 560-nm beam splitter, 510- to 540-nm band pass filter for the green fluorescence detector and 610-nm long pass filter for the red fluorescence detector. The level of background fluorescence, because of the non-specific binding of the FITC-conjugated antibodies, was established using control cell

© Cancer Research Campaign 1998

**Figure 2** Dual-parameter cytograms of DNA content (abscissa) versus FITC-immunolabelling (ordinate) in control (left column) and 10 days ATRA-treated (right column) MCF-7 cells. In each column, the graphs for BrdU (**A**), Ki-67 (**B**), and statin (**C**) immunolabelling are reported. PI-DNA contents and FITC-immunolabellings are expressed in arbitrary units of fluorescence intensity. After treatment with ATRA, there was a significant decrease in the frequency of both S-phase (BrdU-positive) cells and Ki-67 positive cells, whereas the frequency of statin positive cells increased only slightly specimens processed as previously described, but either without incubation with the primary antibodies or with incubation with a mouse serum containing no specific antigenic activity. The corresponding value of green fluorescence was used as a cut-off value above which cells were considered as being labelled. Dual-parameter cytometric data were evaluated with rectangular region analysis: FITC-immunolabelled cells were those with green fluorescence values exceeding the background threshold determined as reported above; the ranges for $G_0$-$G_1$, S- and $G_2$-M phase cells were established on the basis of the corresponding DNA content histograms. At least 20 000 cells per sample were considered in the gated region used for calculations.

**Figure 3** Changes in the frequency of Ki-67-positive and statin-positive cells after different times of treatment with ATRA

### Flow cytometric analysis of apoptotic cells

### *DNA content evaluation*

For DNA staining and flow cytometric analysis, the single-step staining procedure previously described by Pellicciari et al (1993) was used. Either control or ATRA-treated MCF-7 cells were harvested by mild trypsinization and resuspended in complete medium (at a final concentration of $10^6$ cells); 1-ml aliquots were dropped directly into 2 ml of 75 μg $ml^{-1}$ PI solution in water, containing 100 units $ml^{-1}$ of RNAase type A (Sigma Chemical), 10 μM EDTA (to inactivate endogenous endonucleases) and 0.015% Nonidet P40 (Np40 detergent is used here to ensure that both normal and apoptotic cells can be stained). After 60 min of staining, cells were analysed with a FACStar flow cytometer, as reported above. At least 20 000 cells were measured. Values of PI fluorescence were presented as DNA histograms, in which apoptotic cells were identified by the presence of a sub-$G_1$ peak.

### *Labelling with FITC-conjugated Annexin V*

About $10^6$ cells were labelled for 30 min with FITC-conjugated Annexin V (Bender Med-Systems, Prodotti Gianni, Italy) in culture medium (final culture concentration 0.1–1 μg $ml^{-1}$). Cells were then counterstained with a 5 μg $ml^{-1}$ PI solution in the same medium with no detergent; in these conditions, PI stains necrotic (or late apoptotic) cells only, while being excluded by intact (both normal and early apoptotic) cells. Bivariate measurements of green fluorescence (identifying Annexin-labelled cells) vs red fluorescence (identifying cells with damaged membranes) were made with the FACStar flow cytometer.

### RESULTS

DNA content histograms after PI staining are shown in Figure 1. Starting from 3 days of treatment with ATRA, the frequency of MCF-7 cells in the S-phase range of DNA content gradually decreased, and cells accumulated in $G_0/G_1$. The reduced frequency of S-phase cells was confirmed by the incorporation of BrdU. Dual-parameter flow cytograms (Figure 2A) showed a drastic decrease of labelled cells from about 36% in control to about 7% after 10 days of treatment.

Cytometric measurements of DNA content vs Ki-67 labelling (Figure 2B) showed that ATRA had already induced a decrease in the frequency of labelled cells at 24 h of treatment; by 8–10 days of treatment, only 20% of the cells were Ki-67 positive (Figure 3).

© Cancer Research Campaign 1998

**Figure 4** Dual-parameter flow cytograms of FITC-labelled Annexin-V (in abscissa) versus PI staining in isotonic conditions (in ordinate) in MCF-7 cells before (A) and after 1, 2, 3, 5, and 10 days treatment with ATRA (B to F). Normal, non-apoptotic cells were negative for both FITC and PI (quadrant 1); relatively early apoptotic cells were labelled by Annexin V, while being negative for PI (quadrant 2); late apoptotic as well as secondary necrotic cells were labelled both for Annexin V and PI (quadrant 3). Starting with 3 days treatment, the percentage of apoptotic cells progressively increased (3%, 15%, and 22% at 3, 5 and 10 days, respectively); secondary necrotic cells also increased from about 20 to 30%

The percentage of statin-positive cells increased only slightly at 10 days of treatment with ATRA (about 5%, compared with 2% in untreated samples; Figure 2C); this suggests that, at the ATRA concentration used and for the durations of treatment tested, most of the MCF-7 cells accumulated in the $G_1$ phase rather than entering a quiescent $G_0$ state (Figure 3).

Starting from 3 days, a sub-$G_1$ peak was found in DNA histograms (Figure 1), which demonstrates that apoptosis also occurs.

This cytometric evidence was confirmed by the experiments of Annexin V labelling (Figure 4). In early stages of apoptosis, dramatic changes in the organization of plasma membrane take place, among which is the translocation of phosphatidylserine (PS) residues (which, in non-apoptotic cells, lay in the inner phospholipidic leaflet) to the outer surface of apoptotic cells (Martin et al, 1995). Relatively early apoptotic MCF-7 cells therefore become positive for Annexin V, while being negative for PI, which is excluded from the cells; this was apparent in fluorescent and phase-contrast microscopy (data not shown). Biparametric cytograms (Figure 4) showed that, starting at 3 days of treatment, the percentage of apoptotic cells progressively increased (see legend of Figure 4), so that at 10 days of treatment the percentage of dead cells (i.e. apoptotic plus secondary necrotic cells) was about 50%.

### DISCUSSION

It has already been reported that ATRA is able to induce differentiation both in normal and in malignant cells in culture (Breitman et al, 1980). Recently, it has been demonstrated that ATRA may have a strong therapeutic activity in acute promyelocytic leukaemia; its mechanism primarily relates to the property of determining the maturation of the non-differentiated leukaemic promyelocytes into non-proliferating, differentiated granulocytes (Huang et al, 1988; Bollag and Holdener, 1992).

In the rapidly expanding field of clinical oncology, retinoids have also been indicated as being potentially useful agents in different solid tumours, such as breast cancer (Warrel, 1994). ATRA was found to decrease cell growth of MCF-7 cells (Toma et al, 1997). This was confirmed in the present paper by cytometric data, after DNA content evaluation and BrdU vs DNA dual-parameter measurements.

Using single-parameter DNA flow cytometry, however, $G_1$ and $G_0$ cells cannot be discriminated, and it is therefore unclear whether the effect of ATRA on cell cycle progression is due to a lengthening of the $G_1$, pre-DNA synthetic phase and/or to the exit of cells from the cycling compartment into an ‘out of cycle’ quiescent state. The immunolabelling with specific monoclonal antibodies of either the antigen Ki-67 or statin provided the basis

© Cancer Research Campaign 1998

for a more refined cytometric analysis of the cytokinetic effects of ATRA.

The flow cytometric study of cell cycle-related protein vs DNA content provides new landmarks that can be used to subdivide the cell cycle into several distinct subcompartments. The antigen Ki-67 is a non-histone nuclear protein of unknown function, although its direct role in the maintenance of cell proliferation has been demonstrated by the inhibition of DNA synthesis by specific anti-sense oligonucleotides for Ki-67 mRNA (Schluter et al, 1993). This antigen was shown to be absent in $G_0$ cells, while being present in proliferating cells. Ki-67 is expressed at relatively low levels in $G_1$ and early S-phase; it rapidly accumulates during the second half of S-phase, reaching a peak at $G_2$/M phases and decreases sharply in post-mitotic cells (Gerdes, 1984; Bruno and Darzynkiewicz, 1990). Being expressed in all the cycle phases, Ki-67 is a particularly suitable marker for proliferating cells (Gerdes et al, 1984; Schluter et al, 1993), and it may therefore be preferred in cell kinetic studies to other proliferation markers, such as cyclins, whose expression is limited to well-defined phases or even subphases of the cycle (Gong et al, 1994).

Statin is a 57-kDa envelope-associated nuclear protein expressed by quiescent cells of normal or tumour origin both in vivo and in culture (Wang, 1985; Pellicciari et al, 1991; Tsanaclis et al, 1991; Mitmaker et al, 1993). This protein is present in resting $G_0$ cells, and its expression declines when cells re-enter $G_1$, early before the transition into S-phase. The immunolabelling with anti-statin monoclonal antibodies is therefore a unique technique for identifying quiescent cells by light microscopy or by flow cytometry, as we recently demonstrated (Pellicciari et al, 1995).

Using these different cell cycle-related proteins as markers of proliferation potential, we demonstrated that ATRA exerts its antiproliferative effect on MCF-7 cells in culture by blocking most of the cells in early $G_1$ phase of the cell cycle, a few cells only entering $G_0$.

Prolonged (at least 3 days) treatment with ATRA may induce apoptotic cell death in MCF-7 cells; this was confirmed by labelling with Annexin V. In DNA histograms, the presence of a sub-$G_1$ peak demonstrated that apoptosis mostly occurred in the $G_1$ phase, although we cannot exclude, based on the present cytometric evidence, that apoptotic death could also take place in other cell cycle phases. Taken together, these data open interesting perspectives in the study of the relationship between induction of quiescence and commitment of apoptosis in response to anti-neoplastic agents, as it has been recently underlined for retinoic acid in breast cancer (Liu et al, 1996).

Our results indicating that ATRA-induced growth inhibition is associated with specific changes in cell cycle progression of MCF-7 cells should be taken into account when attempting to design treatment programmes that associate ATRA with antineoplastic compounds of different cell cycle specificity.

### ACKNOWLEDGEMENTS

The authors wish to thank Dr Eugenia Wang (The Bloomfield Centre for Aging, Lady Davis Institute for Medical Research, Jewish General Hospital, Montreal, Quebec, Canada) for the kind gift of anti-statin antibody. This study was partly supported by CNR (ACRO grant 92.02252.PF39), by AIRC and by MURST (40% and 60%) grants. Thanks are also due to Dr G Dastoli (Roche, Milan, and G.O.S.R., Study Group for Retinoids in Oncology) for discussion and continuous support. Flow cytometric measurements were performed at the 'Centro Grandi Strumenti' of the University of Pavia.

### REFERENCES

Benbrook P, Lernhardt E and Pfaul M (1988) A new retinoic acid receptor identified from hepatocarcinoma. *Nature* **332**: 669–672

Bollag W and Holdener EE (1992) Retinoids in cancer prevention and therapy. *Ann Oncol* **3**: 513–526

Breitman TR, Selonick SE and Collins SJ (1980) Induction of differentiation of the human promyelocytic leukemia cell line (HL-60) by retinoic acid. *Proc Natl Acad Sci USA* **77**: 2936–2940

Bruno S and Darzynkiewicz Z (1992) Cell cycle dependent expression and stability of the nuclear protein detected by Ki-67 antibody in HL-60 cells. *Cell Prolif* **25**: 31–40

Connor MJ (1986) Retinoid stimulation of epidermal differentiation in vivo. *Life Sci* **38**: 1807–1812

De Luca CM (1991) Retinoids and their receptors in differentiation, embryogenesis and neoplasia. *FASEB J* **45**: 2924–2933

Evans RM (1988) The steroid and thyroid hormone receptor superfamily. *Science* **240**: 889–895

Fontana JA (1987) Interaction of retinoids and tamoxifen on the inhibition of human mammary carcinoma cell proliferation. *Exp Cell Biol* **55**: 136–144

Fontana JA, Hiksis G, Miranda DM and Durham JP (1987) Inhibition of human mammary carcinoma cell proliferation by retinoids and intracellular c-AMP elevating compounds. *J Natl Cancer Inst* **78**: 1107–1112

Fuchs E and Green H (1981) Regulation of terminal differentiation of cultured human keratinocytes by Vitamin A. *Cell* **25**: 617–625

Gerdes S, Lemke H, Baisch H, Wacker MH, Schwab U and Stein H (1984) Cell cycle analysis of a cell-proliferation associated human nuclear antigen defined by the monoclonal antibody Ki-67. *J Immunol* **133**: 1710–1715

Giguere V, Ong ES, Sequi P and Evans RM (1987) Identification of a receptor for the morphogen retinoic acid. *Nature* **330**: 624–629

Gong J, Li X, Traganos F and Darzynkiewicz Z (1994) Expression of G1 and G2 cyclins measured in individual cells by multiparameter flow cytometry: a new tool in the analysis of the cell cycle. *Cell Prolif* **27**: 357–371

Huang H, Ye Y, Chen S, Chai J, Zhoa L, Gu L and Wang Z (1988) Use of all-trans retinoic acid in the treatment of acute promyelocytic leukemia. *Blood* **72**: 567–572

Kopan RG, Trasker G and Fuchs E (1987) Retinoids as important regulators of terminal differentiation: examining keratin expression in individual epidermal cells at various stages of keratinization. *J Cell Biol* **105**: 427–440

Krust A, Kastner P, Petrovich M, Zelent A and Champon P (1989) A third human retinoic acid receptor, h-RAR gamma. *Proc Natl Acad Sci USA* **86**: 5310–5314

Lippman SM, Parkinson DK, Itri LM, Weben RS, Schantw SP, Ota DM, Schusterman MA, Krakoff RS, Gutterman JU and Hong WK (1992) 13-cis retinoic acid and interferon alfa-2a effective combination therapy for advanced squamous cell carcinoma of the skin. *J Natl Cancer Inst* **84**: 235–241

Liu Y, Lee MO, Wang HG, Li Y, Hashimoto Y, Klaus M, Reed JC and Zhang X (1996) Retinoic acid receptor beta mediates the growth-inhibitory effect of retinoic acid by promoting apoptosis in human breast cancer cells. *Mol Cell Biol* **16**: 1138–1149

Martin BS, Reutelingsperger LPM, MC Gahon AJ, Rader JA, Van Schiee RCAA, La Face DM and Green DR (1995) Early redistribution of plasma membrane phosphatidylserine is a general feature of apoptosis regardless of the initiating stimulus: inhibition by overexpression of Bcl-2 and Abl. *J Exp Med* **182**: 1545–1556

Mitmaker B, Bayer I, Bayner S, Gordon PH and Wang E (1993) The differential expression of statin in the nuclei of human colonic crypts adjacent to a cancer: an immunohistochemical study. *Eur J Histochem* **37**: 43–51

Pellicciari C, Danova M, Giordano M, Furhman Conti AM, Mazzini G, Wang E, Ronchetti E, Riccardi A and Manfredi Romanini MG (1991) Expression of cell cycle related proteins proliferating -cell nuclear antigen (PCNA) and statin-during adaptation and de-adaptation of EUE cells to a hypertonic medium. *Cell Prolif* **24**: 469–479

Pellicciari C, Manfredi AA, Bottone MG, Schaack V and Barni S (1993) A single-step staining procedure for the detection and sorting of unfixed apoptotic thymocytes. *Eur J Histochem* **37**: 381–390

© Cancer Research Campaign 1998

Pellicciari C, Mangiarotti R, Bottone MG, Danova M and Wang E (1995) Identification of resting cell by dual parameter flow cytometry of statin expression and DNA content. *Cytometry* **21**: 329–337

Pellicciari C, Bottone MG, Schaack V, Barni S and Manfredi AA (1996) Spontaneous apoptosis of thymocytes is uncoupled with progression through the cell cycle. *Exp Cell Res* **229**: 370–377

Schluter C, Duchrow M, Wohlenberg C, Becker MHG, Key G, Flad Hans-D and Gerder J (1993) The cell proliferation-associated antigen of antibody Ki-67: a very large, ubiquitous nuclear protein with numerous repeated elements, representing a new kind of cell cycle-maintaining proteins. *J Cell Biol* **123**: 513–522

Toma S, Isnardi L, Raffo P, Dastoli G, De Francisci E, Riccardi L, Palumbo R and Bollag W (1997) Effects of All-*trans*-retinoic acid and 13-*cis*-retinoic acid on breast cancer cell lines: growth inhibition and apoptosis induction. *Int J Cancer* **70**: 1–9

Tsanaclis AM, Brem SS, Gately S, Shipper HM and Wang E (1991) Statin immunolocalization in human brain tumors. *Cancer* **33**: 587–594

Wang E (1985) A 57000 mol wt protein uniquely present in non-proliferating cells and senescent human fibroblasts. *J Cell Biol* **100**: 545–551

Wang E and Krueger JG (1985) Application of an unique monoclonal antibody as a marker for non proliferating subpopulations of cells of some tissues. *J Histochem Cytochem* **33**: 587–594

Warrel Jr R (1994) Applications for retinoids in cancer therapy. *Sem Hematol* **31**: 1–13

Wilcken NR, Saracevic B, Musgrave EA and Sutherland RL (1996) Differential effects of retinoids and antiestrogens on cell cycle progression and cell cycle regulatory genes in human breast cancer cells. *Cell Growth Diff* **7**: 65–74

© Cancer Research Campaign 1998